# Addition of immune checkpoint inhibitors to chemotherapy versus chemotherapy alone in patients with triple‐negative breast cancer: A systematic review and meta‐analysis

**DOI:** 10.1002/cam4.6760

**Published:** 2023-12-08

**Authors:** Xin Gao, Ying Zhu, Peipei Wang, Lulin Yu, Shanming Ruan, Minhe Shen, Kai Zhang

**Affiliations:** ^1^ The First Affiliated Hospital of Zhejiang Chinese Medical University (Zhejiang Provincial Hospital of Chinese Medicine) Hangzhou Zhejiang China; ^2^ Anji Traditional Chinese Medical Hospital Huzhou Zhejiang China

**Keywords:** drug therapy, efficacy, immune checkpoint inhibitors, triple‐negative breast cancer

## Abstract

**Background:**

Triple‐negative breast cancer (TNBC) is a relatively common malignant tumor with high mortality rates. There are limited treatment options and current therapy regimens often fall short of providing positive outcomes. The development of immune checkpoint inhibitors (ICIs) have provided a vital treatment option although efficacy has varied. Here, we review patient response to current TNBC treatment with and without the addition of ICIs.

**Methods:**

A systematic search of PubMed, Cochrane, and EMBASE library databases was done to search eligible studies published from their inception through April 3, 2022. The primary outcome indicators used were progression‐free survival (PFS), overall survival (OS), pathological complete response rate (pCR) and objective remission rate (ORR), while adverse events (AEs) were also analyzed. Publication bias and sensitivity analyses and were performed to evaluate the quality of assessment.

**Results:**

Overall, the meta‐analysis looked at seven randomized controlled trials (RCTs) that included 4631 patients with TNBC. Results showed an improvement in PFS for patients receiving ICI in addition to chemotherapy (CT) in both the intent‐to‐treat (ITT) population and PD‐L1 positive patients. Increased pCR rates were observed in all patients irrespective of PD‐L1 status as well as increased ORR in the ITT which was more notable in PD‐L1 positive subjects. While significant improvement in OS was observed only in PD‐L1 positive individuals, the use of ICIs plus CT resulted in severe adverse reactions, specifically immune‐related.

**Conclusions:**

This study supports the increased efficacy of ICIs in combination with CT compared to CT alone in patients with TNBC, with the most notable benefit observed in PD‐L1 positive patients. However, combination therapy increases the risk of adverse reactions which warrants further investigation.

## 1 INTRODUCTION

Breast cancer is a prevalent malignancy with an estimated 43,780 deaths and 290,560 new cases in the United States through 2022.^1^ Triple‐negative breast cancer (TNBC) is one of the main subtypes of breast cancer, accounting for about 20% of all cases, and has the worst prognosis of all subtypes.^2^ Conventional treatments, including general surgery combined with radiotherapy, chemotherapy (CT) and hormone therapy, have not yielded the desired results.^3^ Advancement in understanding the mechanisms of peripheral tolerance, particularly how cancer evades the immune system, has led to the development of immune checkpoint inhibitors (ICIs).^4^ Currently the most common ICIs are Programmed Cell Death Protein 1/Programmed Cell Death Ligand 1 (PD‐1/ PD‐L1) and cytotoxic T‐lymphocyte‐associated protein 4 (CTLA‐4) inhibitors. PDL‐1 or PDL‐2 expressed on tumor cells can bind to the receptor PD1 found on T cells and attenuate T cell activation, proliferation, and cytotoxic secretion leading to impaired anti‐tumor immune responses.^5^ Based on this principle, PD‐1/PD‐L1 antibodies have been developed to preclude this receptor‐ligand interaction and thus increase immune response against cancer cells. Many PD‐1/PDL‐1 drugs, such as pembrolizumab, atezolizumab, and durvalumab, have been approved for various types of cancer and have achieved good clinical efficacy.^6^, ^7^ However, for CTLA‐4 antibody blockade, although it has been shown to enhance antitumor immune responses^8^ and has significant momentum as a therapeutic target,^9^ several questions remain to be addressed. For example, whether the activated T lymphocytes that cause pathology in patients with CTLA‐4 deficiency are antigenic specific remains unclear,^10^ which requires further investigation and understanding of the molecular mechanisms underlying CTLA‐4 and immune disorders. In addition, there are a lack of clinical studies targeting CTLA‐4 in immunotherapy.^11^

Despite the significant therapeutic effects seen in the clinic, ICI drugs can cause immune‐related adverse events (irAEs) that may require suspension or cessation of treatment.^12^ Therefore, knowing which cancer patients will benefit from the addition of ICIs will help limit unwarranted irAEs. For different types of cancer, doctors can choose relatively safe drugs and pay more attention to the changes in the patient's condition,^13^ thus reducing the impact of adverse reactions and improve on provisional treatment.

TNBC has a poor prognosis due in part to the lack of molecular targets for this subtype. However, recent studies have shown that combination immunotherapy has a significant therapeutic effect.^14^, ^15^ Some combination therapies, such as ICI combined with CT, may lead to improvements in the prognosis of TNBC patients, as combination CT generally demonstrates a higher PFS rate than CT alone.^16^ Recent results from randomized controlled trials (RCTs) were consistent with this notion. IMpassion130^17^ and KEYBALL‐355,^18^ both showed that compared with CT alone, patients with ICIs plus CT have longer PFS and overall survival (OS). Furthermore, ICI may have better clinical effects in patients with TNBC that are positive for PD‐L1,^19^ or have high tumor‐infiltrating lymphocytes expression.^20^ Despite these results, IMpassion131,^21^ as well as other studies,^22^, ^23^, ^24^ showed no significant advantage of ICIs in patient OS compared with CT alone. The lack of enhanced OS could be due to acquired resistance to ICIs or disease recurrence after discontinuation of treatment.^25^, ^26^ In addition, ICI combination therapy may lead to an increase in irAEs.^12^ These irAEs may be late onset and persistent,^27^ which may affect treatment results.

Therefore, we performed a systematic review and meta‐analysis of patients with TNBC treated with ICI plus CT and compared clinical outcomes to those treated with CT alone in RCTs. The aim of this study was to analyze the degree of benefit in providing an ICI in combination with CT compared to CT alone in different clinical indicators to guide decision‐making in clinical practice.

## 2 METHODS

The evaluation followed the Preferred Reporting Items for Systematic Reviews and Meta‐Analyses (PRISMA) statement.^28^ The assessment has been registered with PROSPERO (CRD42022328149). The full text can be found on the website (http://www.crd.york.ac.uk/PROSPERO).

### 2.1 Search strategy

Two investigators (Xin Gao and Yin Zhu) independently used PubMed, Cochrane, and EMBASE library databases to search eligible studies from inception to April 3, 2022. Search terms included “PD‐1”, “PD‐L1”, “breast cancer”, “chemotherapy”, and “randomized controlled trials” (Supplementary Text S1). A manual review of the literature was also performed to maximize the search for eligible and relevant articles. When data from the same clinical trial were found in more than one publication, the most recent published data, or the data that was in the publication that was consistent with the search content, was selected.

### 2.2 Eligibility criteria

Two investigators (Xin Gao and Yin Zhu) were responsible for article selection. A third investigator (Peipei Wang) was consulted in the case of any disagreement. The included studies met the following criteria: (a) Patients with malignant breast cancer; (b) Treatment Group: Immune checkpoint inhibitors combined with chemotherapy; (c) Control group: chemotherapy alone, with or without placebo; (d) Categorized as progression‐free survival (PFS), OS, objective remission rate (ORR) or pathological complete response rate (pCR) based study data; (e) Randomized controlled trials; and (f) Sample size of more than 10 patients in each group. Meanwhile, the exclusion criteria were as follows: (a) Patients with no malignant breast cancer; (b) Treatment Group: Did not contain ICIs combined with chemotherapy; (c) Control group: Not chemotherapy alone, with or without placebo; (d) Did not have PFS, OS, ORR or pCR based study data; (e) Not RCTs; (f) Sample size of less than 10 patients in each group.^28^

### 2.3 Quality assessment

Two investigators (Xin Gao and Yin Zhu) independently assessed the risk of bias according to the Cochrane Collaboration's Risk of Bias assessment tool.^29^ The two reviewers solved any inconsistencies through discussion to reach a consensus. Publication bias was also evaluated by a funnel plot. Sensitivity analysis was performed to determine any source of heterogeneity and assess result stability.

### 2.4 Statistical analysis

All data analyses were performed using RevMan software (Windows version 5.3). The combined hazard ratio (HR) and 95% confidence interval (CI) were calculated using the random effects model for FPS and OS, whereas the odds ratio (OR) of association and the corresponding 95% CI were used for pCR, ORR, and adverse events (AE). *p <* 0.05 was considered statistically significant, and all tests were two‐way. According to the degree of heterogeneity *I* ^*2*^, different models were used for aggregation. When *p* > 0.1 and *I* ^*2*^ < 50%, a fixed effects model was used; otherwise, a random effects model was used. A sensitivity analysis of the included literature was performed to identify the source of all heterogeneity and to assess the stability of the results.

## 3 RESULTS

### 3.1 Search results and study characteristics

**FIGURE 1:**
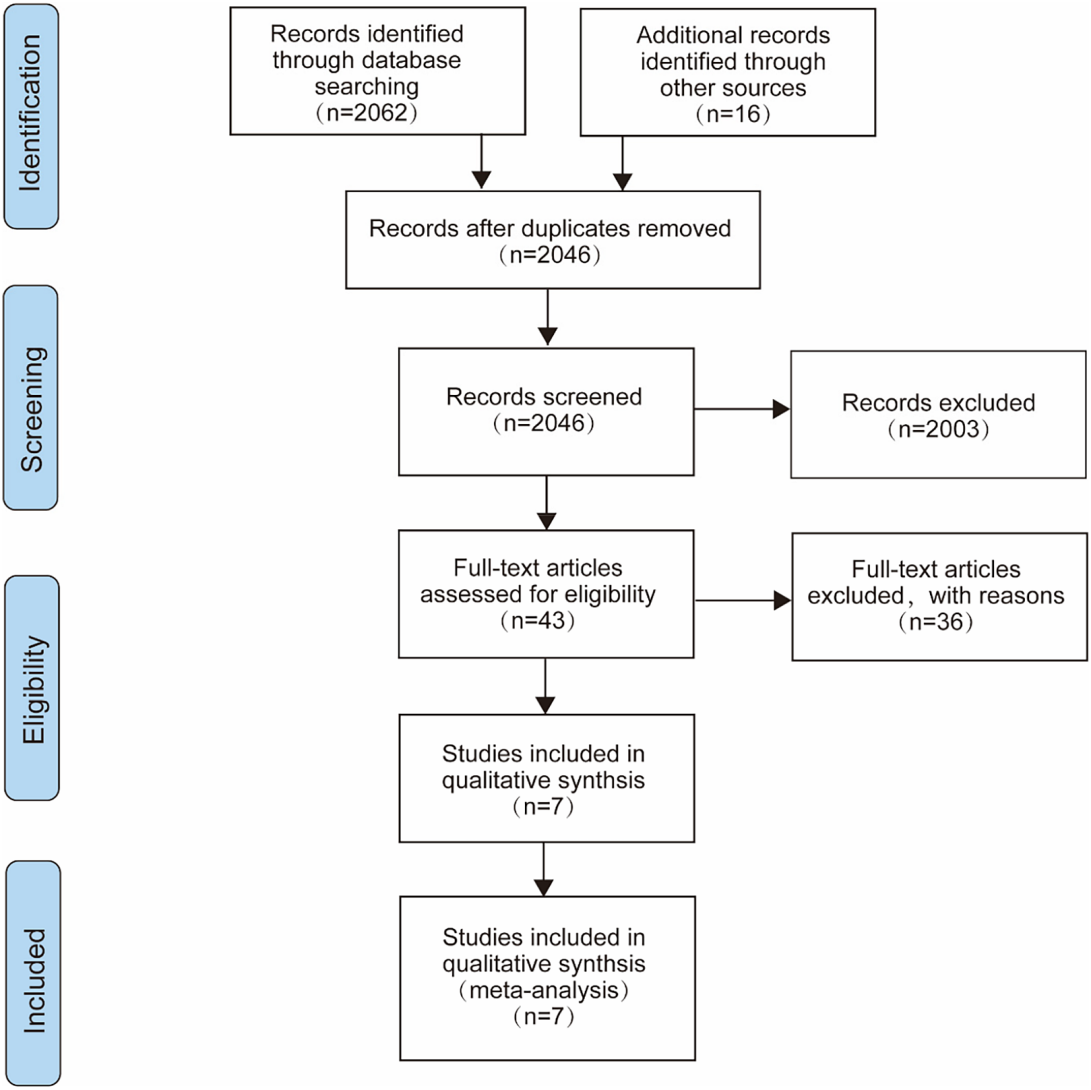
The flow diagram of literature selection.

A total of 2062 articles were retrieved through the adopted search strategy and 16 related reports were retrieved manually. Duplicate articles were eliminated by manual review leaving a total of 2046 articles. From these remaining articles, 2003 were identified as irrelevant, leaving 43 entries for full reading. Seven of these records met the eligibility criteria and were included in the final analysis.^17^, ^18^, ^21^, ^22^, ^23^, ^30^, ^31^ Each step was independently conducted and proofread by two investigators. The study inclusion process is shown in Figure 1.

All included studies were published between 2018 and 2021 and were first‐line treatments. Our meta‐analysis included 4361 patients from the seven RCTs, including 2623 patients in the treatment group and 1738 in the control group (Table 1).

**TABLE 1:** Main characteristics of studies included in the meta‐analysis.

Study	Year	Registration number	Treatment line	Study Phase	Cancer stage	Primary/Secondary endpoint	Case	Chemotherapy drug	ICI drug(target)	Number of PD‐L1 positive
NeoTRIP^22^	2019	NCT002620280	First line	III	Phase II/III TNBC	EFS/pCR	138 vs. 142	Nab‐Pac	Ate (PD‐L1)	NA
GeparNuevo^23^	2019	NCT02685059	First line	II	Phase I/II/III TNBC	pCR/pCR	88 vs. 86	Nab‐Pac	Dur (PD‐L1)	138
IMpassion130^17^	2020	NCT02425891	First line	III	Phase III/IV TNBC	PFS, OS	451vs. 451	Nab‐Pac	Ate (PD‐L1)	369
KEYNOTE‐355^18^	2020	NCT02819518	First line	III	Phase IV TNBC	OS, PFS/ORR, DCR, DOR	566vs. 281	Nab‐Pac, Pac, Car,Gem	Pem (PD‐1)	323
KEYNOTE‐522^31^	2020	NCT03036488	First line	III	Phase II/III TNBC	EFS, pCR/OS	784 vs. 390	Pac, Car, Pem	Pem (PD‐1)	973
IMpassion031^30^	2020	NCT03197935	First line	III	Phase II/III TNBC	pCR/OS, DFS, EFS	165 vs. 168	Nab‐Pac	Ate (PD‐L1)	154
IMpassion131^21^	2021	NCT03125902	First line	III	Phase III/IV TNBC	PFS/OS, ORR	431 vs. 220	Pac	Ate (PD‐L1)	292

Abbreviations: Ate, atezolizumab; Car, carboplatin; DCR, disease control rate; DFS, disease‐free survival; DOR, duration of response; EFS, event‐free survival; Gem, gemcitabine; HR, hazard ratio; ICI, Immune checkpoint inhibitors; NA, not available; Nab‐Pac, nab‐paclitaxel; ORR, objective response rate; OS, overall survival; Pac, Paclitaxel; pCR, pathological complete response; Pem, pembrolizumab; PFS, progression‐free survival.

### 3.2 Progression‐free survival

**FIGURE 2:**
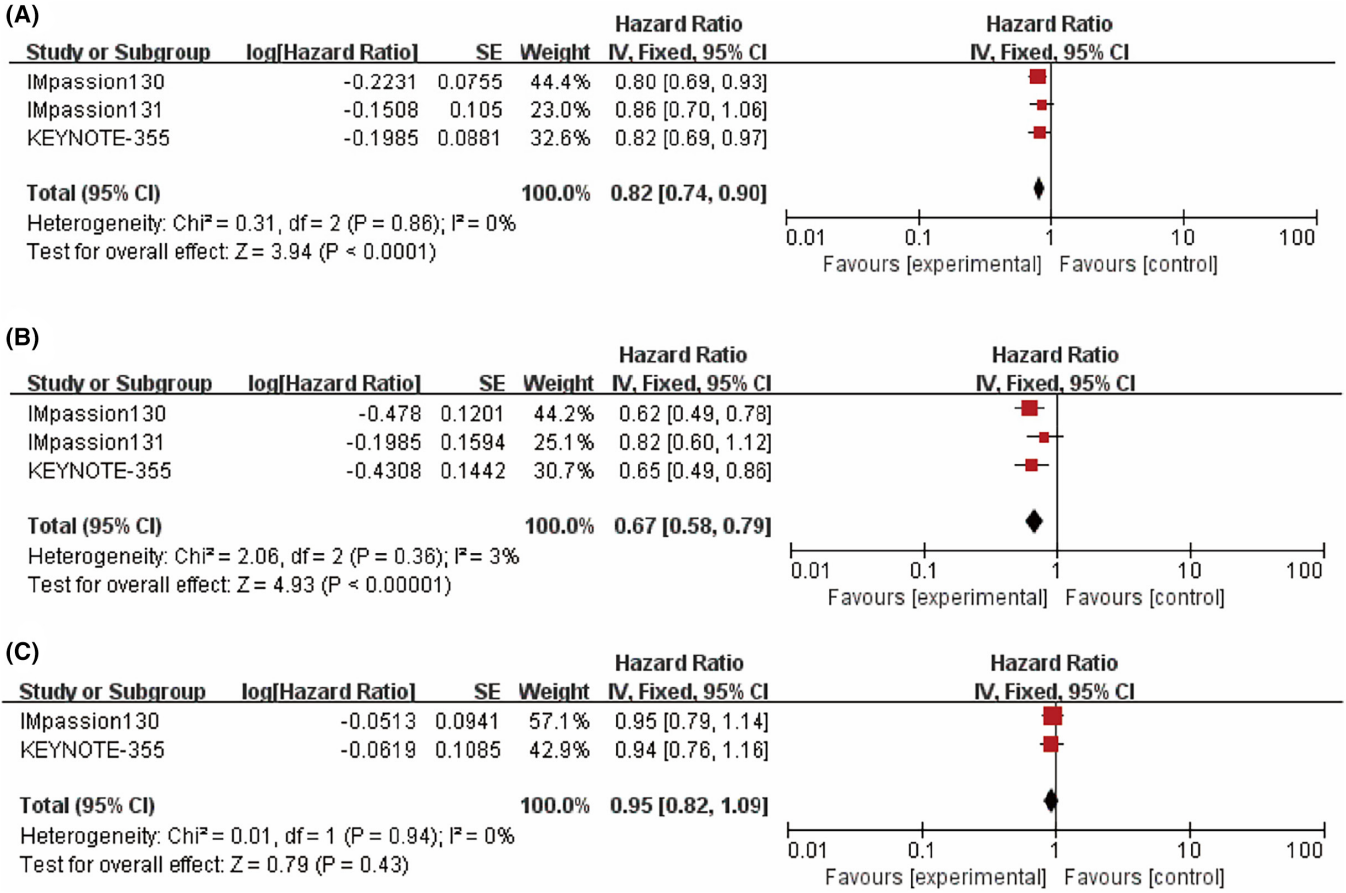
Forest plots comparing progression‐free survival (PFS) hazard ratios between treatment and control groups. (A) Intent‐to‐treat (ITT) patients. (B) PD‐L1 positive patients. (C) PD‐L1 negative patients.

When evaluating PFS, an overall estimate of the ITT population (*n* = 2396) showed the benefit of combining ICIs with CT (HR = 0.82; 95% CI: 0.74–0.90; *p <* 0.0001, Figure 2A) and no heterogeneity was found in the PFS estimates included in the study (*I* ^*2*^ = 0%). However, when taking into account patient PD‐L1 status, only patients positive for PD‐L1 (*n* = 984) showed a significant increase in PFS after ICI treatment (HR = 0.67; 95% CI: 0.58–0.79; *p <* 0.00001, *I* ^*2*^ = 3%, Figure 2B), while PFS in PD‐L1 negative patients was not statistically significant (*p =* 0.43, Figure 2C).

### 3.3 Overall survival

**FIGURE 3:**
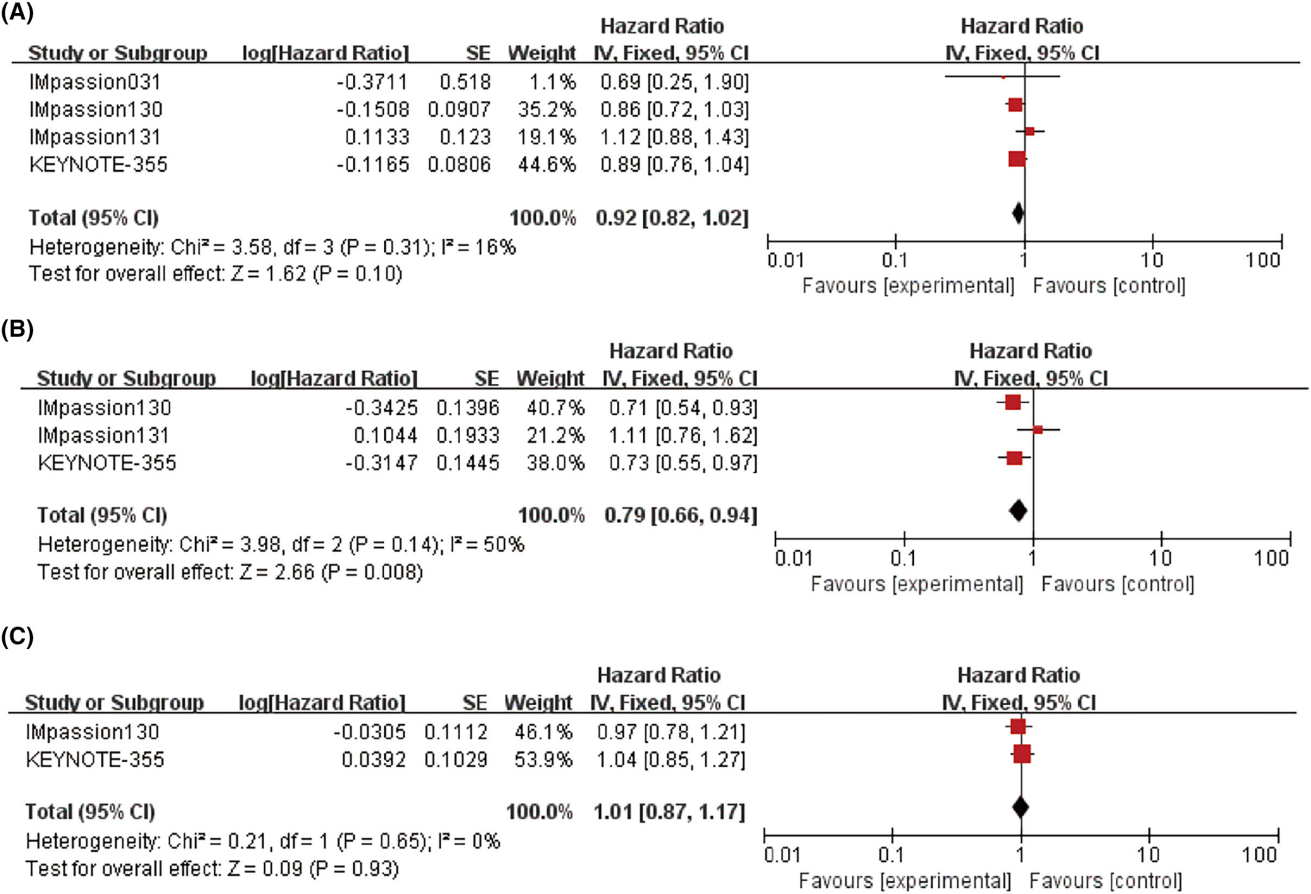
Forest plots comparing overall survival (OS) hazard ratios between treatment and control groups. (A) Intent‐to‐treat (ITT) patients. (B) PD‐L1 positive patients. (C) PD‐L1 negative patients.

In terms of OS (*n* = 2729), a slight improvement was found with the addition of ICI in the ITT population, but this increase was not statistically significant, *p =* 0.10 (HR = 0.92; 95% CI: 0.82–1.02; *I* ^*2*^ = 16%, Figure 3A). However, a significant improvement was observed in patients who were positive for PD‐L1 (*n* = 984) (HR = 0.79; 95% CI: 0.66–0.94; *p =* 0.008; *I* ^*2*^ = 50; Figure 3B). The PD‐L1 negative group did not have a significant improvement in OS with the addition of ICI (*p =* 0.93, Figure 3C).

### 3.4 Pathological complete response rate

**FIGURE 4:**
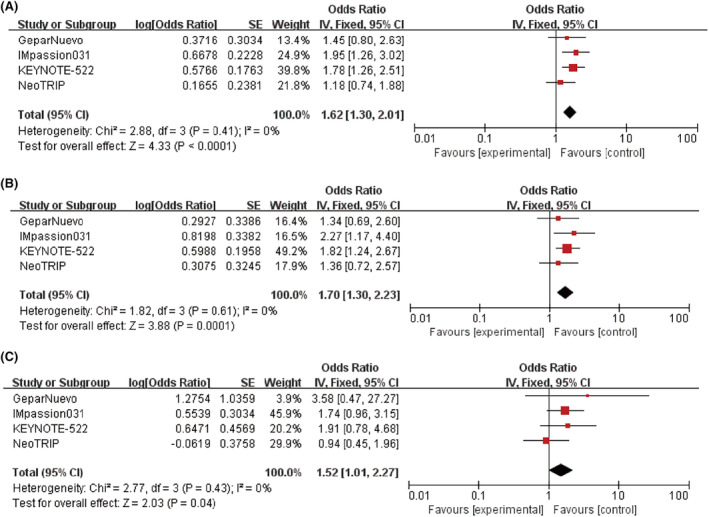
Forest plots of odds ratio (ORs) for pathological complete response rate (pCR) comparing treatment and control group. (A) In the intent‐to‐treat (ITT) patients. (B) PD‐L1 positive patients. (C) PD‐L1 negative patients.

The pCR effect of ICI combined with CT was better than that of CT alone in the ITT population (*n* = 1961) (OR = 1.62, 95% CI: 1.30–2.01; *p <* 0.0001; Figure 4A). This finding was consistent for both PD‐L1 positive patients (OR = 1.70, 95%CI: 1.30–2.23; *p =* 0.0001; *I* ^*2*^ = 0%, Figure 4B) and PD‐L1 negative patients (OR = 1.52, 95% CI: 1.01–2.27; *p =* 0.04; *I* ^*2*^ = 0%; In Figure 4C).

### 3.5 Objective response rate

**FIGURE 5:**
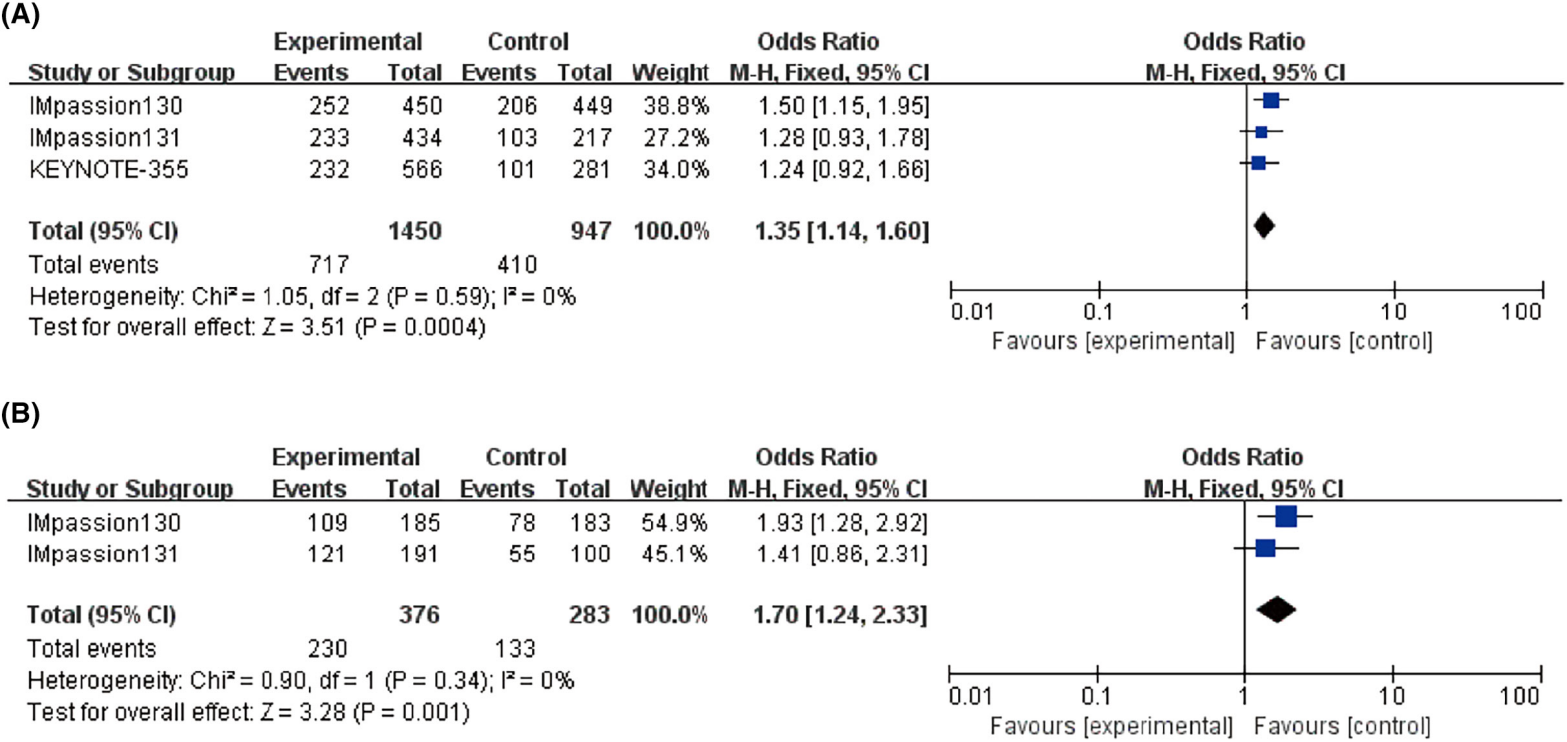
Forest plots of odds ratio (ORs) for objective response rate (ORR) comparing treatment and control group. (A) Intent‐to‐treat (ITT) patients. (B) PD‐L1 positive patients.

The ORR of ICI combined with CT was superior to that of CT alone in the ITT population (*n* = 2397) (OR = 1.35, 95% CI: 1.14–1.60; *I* ^*2*^ = 0%; *p =* 0.0004; Figure 5A) as well as in the PD‐L1 positive patients (OR = 1.70, 95% CI: 1.24–2.23; *p =* 0.001; *I* ^*2*^ = 0%, Figure 5B).

### 3.6 Adverse reactions

**FIGURE 6:**
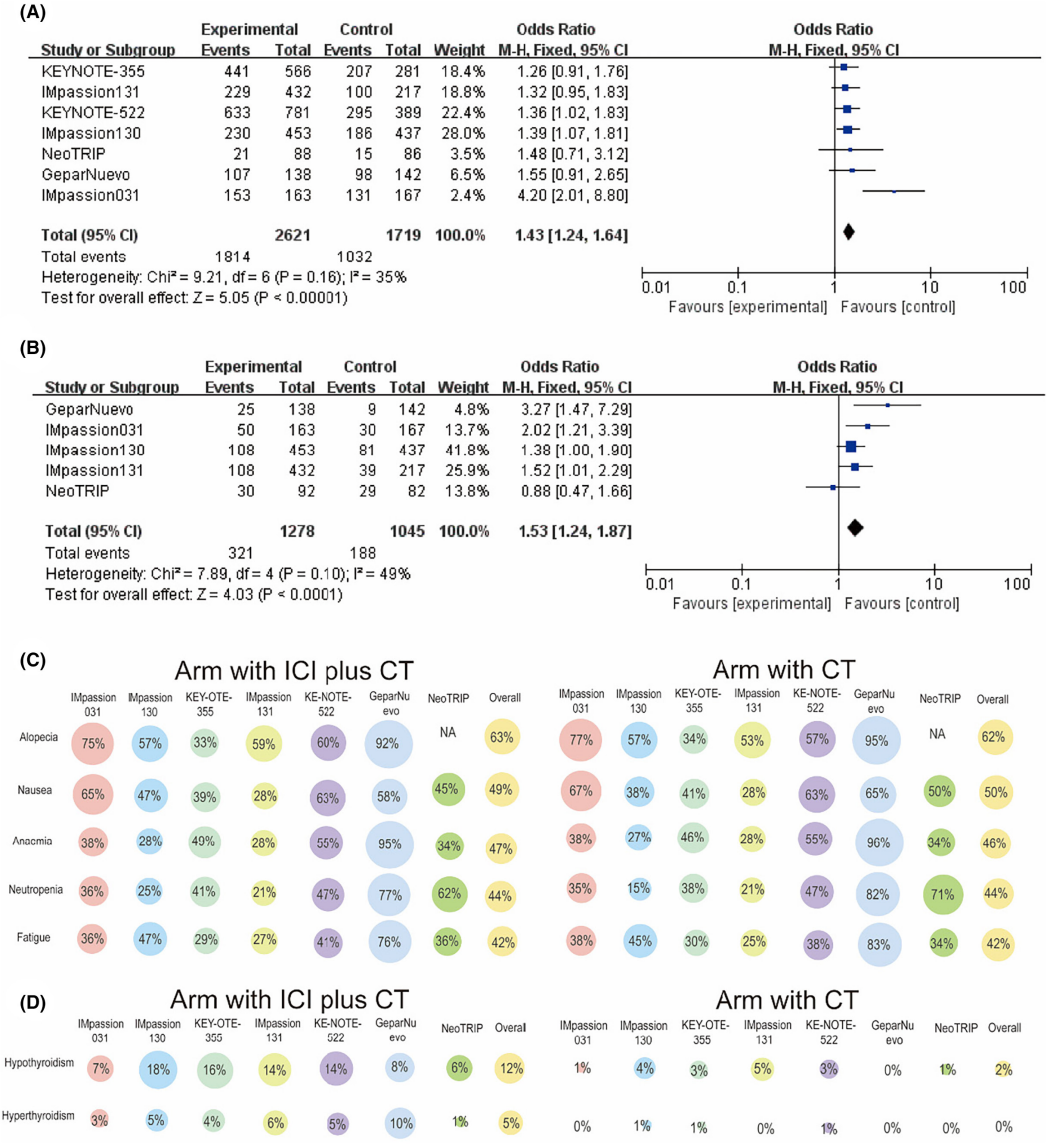
Security analysis. (A) Grades 3–5 AEs observed with ICI combined with CT and CT alone. (B) Severe AEs compared between ICI combined with CT and CT alone. (C) The frequency of the most common AEs for any grade. (D) The frequency of the most common irAEs of any grade.

In the ICI treatment group, 69.2% of patients experienced Grade 3 or greater adverse events compared with 59.5% in the control group. For Grade 3–5 adverse events, an OR of 1.43 (95% CI: 1.24–1.64; *p <* 0.00001; *I* ^*2*^ = 16%, Figure 6A) indicated that adding ICI may increase occurrence probability. In terms of serious adverse events (SAE), the prevalence was 25.1% in the ICI treatment group and 18.0% in the control group, indicating that the incidence of a SAE was also higher with the addition of ICI (OR 1.53, 95% CI: 1.24–1.87; *p <* 0.0001, *I* ^*2*^ = 49%, Figure 6B).

We analyzed the frequency of specific AEs at any level in these nine RCTs. The most common AEs were alopecia (63% in ICIs plus CT vs. 62% in CT alone), nausea (49% in ICIs plus CT vs. 50% in CT alone), anemia (47% in ICIs plus CT vs. 46% in CT alone), neutropenia (44% in ICIs plus CT vs. 44% in CT alone) and fatigue (42% in ICIs plus CT vs. 42% in CT alone). There was no significant difference in the frequency of the most common AEs at any grade between the ICI treated and control groups (Figure 6C). The most common irAEs in the ICI treatment group were hypothyroidism (12%) and hyperthyroidism (5%), while hypothyroidism was less common in the control group (2%) and hyperthyroidism was almost nonexistent (Figure 6D).

### 3.7 Subgroup analysis

**FIGURE 7:**
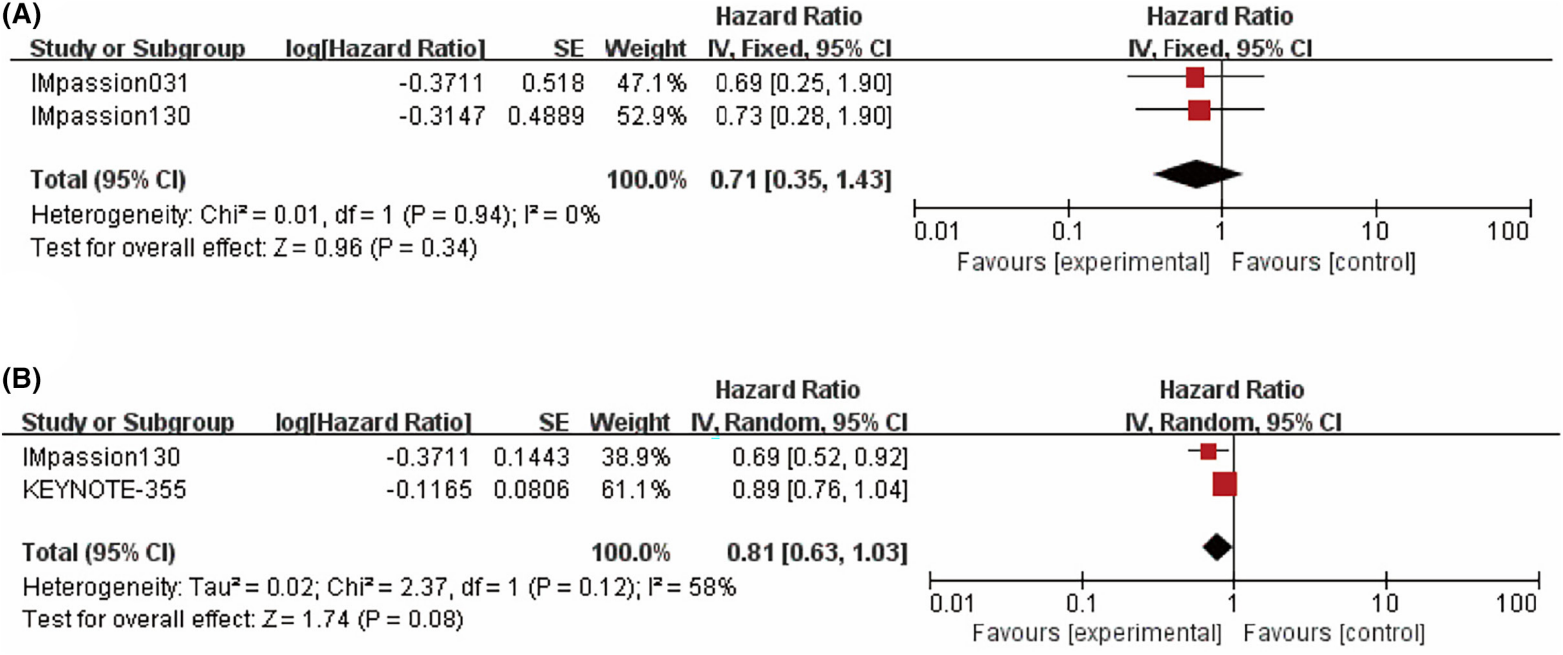
Forest plots comparing overall survival (OS) hazard ratios between treatment and control groups. (A) In Stage I‐III TNBC patients. (B) In Stage IV TNBC patients.

We conducted a corresponding subgroup analysis due to the treatment differences between metastatic and local TNBC. When analyzing OS (*n* = 2082) in Stage I‐III patients and Stage IV patients, the combined treatment effect of ICI and CT was marginally better but did not reach statistical significance (*p* > 0.05) (Figure 7).

### 3.8 Quality assessment

We summarized the quality evaluation of the seven included studies (Figure S1) and found that they had a low risk of bias. Among them, five trials^17^, ^22^, ^23^, ^30^, ^31^ were assessed as uncertain risk of selection bias, and one trial^22^ was considered as skeptical selection bias. In addition, one^31^ trial was evaluated as unclear reporting bias. As for the other bias, two tests^21^, ^30^ were considered as low risk, while the other tests were unclear risk of bias.

### 3.9 Publication bias test and sensitivity analysis

The acceptable asymmetry is illustrated in the funnel plot and indicated a low risk of publication bias (Figure S2). Sensitivity analysis was performed by converting the random effects model into the fixed effects model and showed that the results did not significantly alter (Table S1). The exclusion of every study demonstrated minor change in the pooled risk of the primary outcome which proved that the results were relatively stable (Table S2).

## 4 DISCUSSION

To the best of our knowledge, this is the latest comprehensive meta‐analysis of CT alone versus CT and ICIs in the treatment of TNBC patients. Our results indicate that combination therapy may have better clinical outcomes, particularly for those patients who are PD‐L1 positive.

Our analysis showed that in PFS, ITT (HR = 0.82; 95% CI: 0.74–0.90) and PD‐L1 positive (HR = 0.67; 95%CI: 0.58–0.79) patients benefit from combination therapy, and PD‐L1 positive patients were significantly better than ITT population. Only PD‐L1 positive patients showed an improvement in OS when given an ICI in combination with CT (HR = 0.79; 95% CI: 0.66–0.94). However, when looking at pCR, the ITT population (OR = 1.62, 95% CI: 1.30–2.01), along with both PD‐L1 positive patients (OR = 1.70, 95%CI: 1.30–2.23) and PD‐L1 negative patients (OR = 1.52, 95% CI: 1.30–2.23) showed that combination therapy was superior to CT alone. Likewise, ORR was significantly better in the ITT (OR = 1.35, 95% CI: 1.14–1.60) and PD‐L1 positive patients (OR = 1.70, 95% CI: 1.24–2.23) when given ICIs. ICI plus CT resulted in more AEs (OR = 1.43; 95% CI 1.24–1.64) and SAEs (OR = 1.53, 95% CI 1.24–1.87) than CT given alone. In addition, ICIs led to more irAE. These results indicate that combined therapy has a better therapeutic effect than CT alone but that patients given ICIs need to be monitored for AEs. In our study, populations with PD‐L1 positive in OS had high degree heterogeneity (*I* ^2^ = 50%). This may be due to the use of different CT drugs, different ICIs drugs, or inconsistent definitions of PD‐L1 positive people. While not statistically significant, subgroup analyses showed that patients with both localized and metastatic TNBC benefited from combination therapy. This result suggests that adding ICI to CT may benefit TNBC patients to some extent regardless of disease stage. This benefit is more prominent in PD‐L1 positive patients which holds clinical significance for selecting patient treatment options. At the same time, we believe that it is necessary to evaluate PD‐L1 in TNBC patients, which will facilitate the selection of patients who will benefit more from combination therapy. However, due to the increased risk of AEs, it is necessary for clinicians to monitor patients closely when administering ICIs and make adjustments when necessary.

One thought in the field is that CT alone may cause drug resistance in tumor cells which would lead to a reduced therapeutic effect.^32^ However, here we show that the ICI treatment group has better outcomes than the control group, which might be because the combination therapy had subadditive, additive or synergistic effects.^33^, ^34^ PD‐1 binding to PD‐L1 regulates the autoimmune system by inhibiting T cell activity and inducing T cell apoptosis.^35^ Tumor cells evade immune surveillance by overexpressing PD‐L1,^36^ which provides a scientific basis for the clinical application of ICI in oncology. In our meta‐analysis, PD‐L1 positive patients had significantly better PFS and OS than PD‐L1 negative patients. Similar conclusions were obtained in some of the included studies, such as IMpassion130, Keynote‐355, and Keynote‐522, which may be because PD‐L1 positive patients are more sensitive to ICI drugs.^37^ However, IMpassion131 results were inconsistent which might be attributed to differing CT drugs and CT regiments^38^ or might be related to varying proportions of random allocation and experimental errors. Nonetheless, our analysis confirmed that ICI plus CT can improve the short‐term pCR rate of patients with TNBC compared with CT alone, as well as improve the ORR of ITT patients and PD‐L1 positive patients. This finding suggests that combination therapy is beneficial for patients with TNBC compared to CT alone.

We evaluated patients with Grade 3–5 AE and SAE and showed that adding ICIs in combination with CT led to an increased risk of adverse reactions. Our statistics showed no significant difference between the ICI treatment group and the control group for any level of specific AEs such as hair loss, nausea, fatigue, anemia, and leukopenia. However, for some immune‐related adverse reactions, such as hypothyroidism and hyperthyroidism, the incidence in the ICI treatment group (12% and 5%, respectively) was much higher than that of the control group (2% and 0%, respectively), which is consistent with other studies.^39^, ^40^ It has been suggested that there may be a positive correlation between immune‐related side effects and the efficacy and benefits of ICIs.^41^ In addition, the recurrence rate of adverse events is high, with between one‐quarter and one‐third of patients experiencing the same adverse events after resuming ICI treatment.^42^ Although ICI has extensive and lasting safety,^43^ clinical monitoring of adverse reactions should also be emphasized when ICIs are added to CT.

Limitations in our study should be noted. First, our analysis is based on a literature search which leads to some deviation in statistical results.^44^ Second, PD‐L1 expression has some limitations due to the different detection methods used in the various studies.^45^ Third, significant heterogeneity was found in OS and SAE analyses, which may reduce the reliability of the statistical results. Finally, the data from some of the RCTs were not comprehensive. For example, IMpassion031 did not report patient PFS and Keynote‐522 did not report patient OS, which may lead to some limitations in the experimental results.

## 5 CONCLUSION

Our meta‐analysis showed that ICI combined with CT significantly improved PFS in the ITT population as well as patients with PD‐L1 positive TNBC compared to CT treatment alone. However, only PD‐L1 positive patients had improved OS with the addition of ICI. Importantly, there was increased pCR rates in all patients and increased ORR in ITT and PD‐L1 positive subjects. Nevertheless, despite the benefits seen with the addition of ICIs, there is an increased risk of both AE and irAE that should be taken into consideration.

## AUTHOR CONTRIBUTIONS

**Xin Gao:** Data curation (equal); formal analysis (equal); investigation (equal); writing – original draft (equal). **Ying Zhu:** Project administration (equal); writing – review and editing (equal). **Peipei Wang:** Investigation (equal). **Lulin Yu:** Project administration (equal). **SHANMING RUAN:** Methodology (equal). **Minhe Shen:** Supervision (equal). **Kai Zhang:** Conceptualization (lead); resources (supporting).

## CONFLICT OF INTEREST STATEMENT

The authors declare no conflict of interest.

## Supporting information


Data S1:

## Data Availability

The data that support the findings of this study are openly available in NCBI (https://www.ncbi.nlm.nih.gov/).
