# Supplementary material for: Addition of immune checkpoint inhibitors to chemotherapy versus chemotherapy alone in patients with triple‐negative breast cancer: A systematic review and meta‐analysis
Source: Cancer Med. 2023 Dec 8;12(24):21873–84. doi: 10.1002/cam4.6760 (PMC10757081; doi:10.1002/cam4.6760)
Supplement: Supplementary file 1 — Data S1: [file CAM4-12-21873-s001.docx]

Addition of immune checkpoint inhibitors to chemotherapy versus chemotherapy alone in patients with triple-negative breast cancer: A systematic review and meta-analysis

*Supplementary materials*

**Supplementary Text S1**. The specific retrieval strategy in each database.

1. PubMed retrieval strategy

((((breast cancer[MeSH Terms]) OR (Breast Neoplasm OR Neoplasm, Breast OR Breast Tumors OR Breast Tumor OR Tumor, Breast OR Tumors, Breast OR Neoplasms, Breast OR Breast Cancer OR Cancer, Breast OR Mammary Cancer OR Cancer, Mammary OR Cancers, Mammary OR Mammary Cancers OR Malignant Neoplasm of Breast OR Breast Malignant Neoplasm OR Breast Malignant Neoplasms OR Malignant Tumor of Breast OR Breast Malignant Tumor OR Breast Malignant Tumors OR Cancer of Breast OR Cancer of the Breast OR Mammary Carcinoma, Human OR Carcinoma, Human Mammary OR Carcinomas, Human Mammary OR Human Mammary Carcinomas OR Mammary Carcinomas, Human OR Human Mammary Carcinoma OR Mammary Neoplasms, Human OR Human Mammary Neoplasm OR Human Mammary Neoplasms OR Neoplasm, Human Mammary OR Neoplasms, Human Mammary OR Mammary Neoplasm, Human OR Breast Carcinoma OR Breast Carcinomas OR Carcinoma, Breast OR Carcinomas, Breast))) AND ((chemotherapy[MeSH Terms]) OR (Therapy, Drug OR Drug Therapies OR Therapies, Drug OR Chemotherapy OR Chemotherapies OR Pharmacotherapy OR Pharmacotherapies))) AND ((Immune checkpoint inhibitors[MeSH Terms]) OR (Immune checkpoint inhibitors or Checkpoint Inhibitors, Immune or Immune Checkpoint Inhibitor or Checkpoint Inhibitor, Immune or Immune Checkpoint Blockers or Checkpoint Blockers, Immune or Immune Checkpoint Blockade or Checkpoint Blockade, Immune or Immune Checkpoint Inhibition or Checkpoint Inhibition, Immune or PD-L1 Inhibitors or PD L1 Inhibitors or PD-L1 Inhibitor or PD L1 Inhibitor or Programmed Death-Ligand 1 Inhibitors or Programmed Death Ligand 1 Inhibitors or CTLA-4 Inhibitors or CTLA 4 Inhibitors or CTLA-4 Inhibitor or CTLA 4 Inhibitor or Cytotoxic T-Lymphocyte-Associated Protein 4 Inhibitors or Cytotoxic T Lymphocyte Associated Protein 4 Inhibitors or Cytotoxic T-Lymphocyte-Associated Protein 4 Inhibitor or Cytotoxic T Lymphocyte Associated Protein 4 Inhibitor or PD-1 Inhibitors or PD 1 Inhibitors or PD-1 Inhibitor or Inhibitor, PD-1 or PD 1 Inhibitor or Programmed Cell Death Protein 1 Inhibitor or Programmed Cell Death Protein 1 Inhibitors or PD-1-PD-L1 Blockade or Blockade, PD-1-PD-L1 or PD 1 PD L1 Blockade))

1. Cochrane Library retrieval strategy

#1 MeSH descriptor: [Breast Neoplasms] explode all trees 14489

#2 Breast Neoplasm OR Neoplasm, Breast OR Breast Tumors OR Breast Tumor OR Tumor, Breast OR Tumors, Breast OR Neoplasms, Breast OR Breast Cancer OR Cancer, Breast OR Mammary Cancer OR Cancer, Mammary OR Cancers, Mammary OR Mammary Cancers OR Malignant Neoplasm of Breast OR Breast Malignant Neoplasm OR Breast Malignant Neoplasms OR Malignant Tumor of Breast OR Breast Malignant Tumor OR Breast Malignant Tumors OR Cancer of Breast OR Cancer of the Breast OR Mammary Carcinoma, Human OR Carcinoma, Human Mammary OR Carcinomas, Human Mammary OR Human Mammary Carcinomas OR Mammary Carcinomas, Human OR Human Mammary Carcinoma OR Mammary Neoplasms, Human OR Human Mammary Neoplasm OR Human Mammary Neoplasms OR Neoplasm, Human Mammary OR Neoplasms, Human Mammary OR Mammary Neoplasm, Human OR Breast Carcinoma OR Breast Carcinomas OR Carcinoma, Breast OR Carcinomas, Breast 42715

#3 #1 OR #2 42715

#4 MeSH descriptor: [Drug Therapy] explode all trees 147649

#5 Therapy, Drug OR Drug Therapies OR Therapies, Drug OR Chemotherapy OR Chemotherapies OR Pharmacotherapy OR Pharmacotherapies 522280

#6 #4 OR #5 558623

#7 MeSH descriptor: [Immune Checkpoint Inhibitors] explode all trees 70

#8 Bavencio OR Tecentriq OR Libtayo OR Opdivo OR Keytruda OR avelumab OR sintilimab OR toripalimab OR cemiplimab OR durvalumab OR atezolizumab OR pembrolizumab OR Tremelimumab OR Ipilimumab 5871

#9 #7 OR #8 5900

#10 #3 AND #6 AND #9 421

1. Cochrane Library retrieval strategy

#8 #5 AND #6 AND #7

576

#7 'chemotherapy'/exp OR 'therapy, drug'/exp OR 'pharmacotherapy'/exp

3,247,201

#6 'breast cancer'/exp OR 'breast tumor'/exp OR 'breast neoplasms'/exp

626,414

#5 #3 AND #4

6,644

#4 'randomized controlled trial'/exp OR 'randomization'/exp OR 'double blind procedure'/exp OR 'placebo'/exp

1,071,426

#3 #1 OR #2

94,778

#2 'programmed death 1 receptor'/exp OR 'nivolumab'/exp OR 'pembrolizumab'/exp OR 'atezolizumab'/exp OR 'durvalumab'/exp OR 'cemiplimab'/exp OR 'toripalimab'/exp OR 'sintilimab'/exp OR 'avelumab'/exp OR 'camrelizumab'/exp

73,052

#1 'ticilimumab'/exp OR 'ipilimumab'/exp OR 'ctla 4'/exp


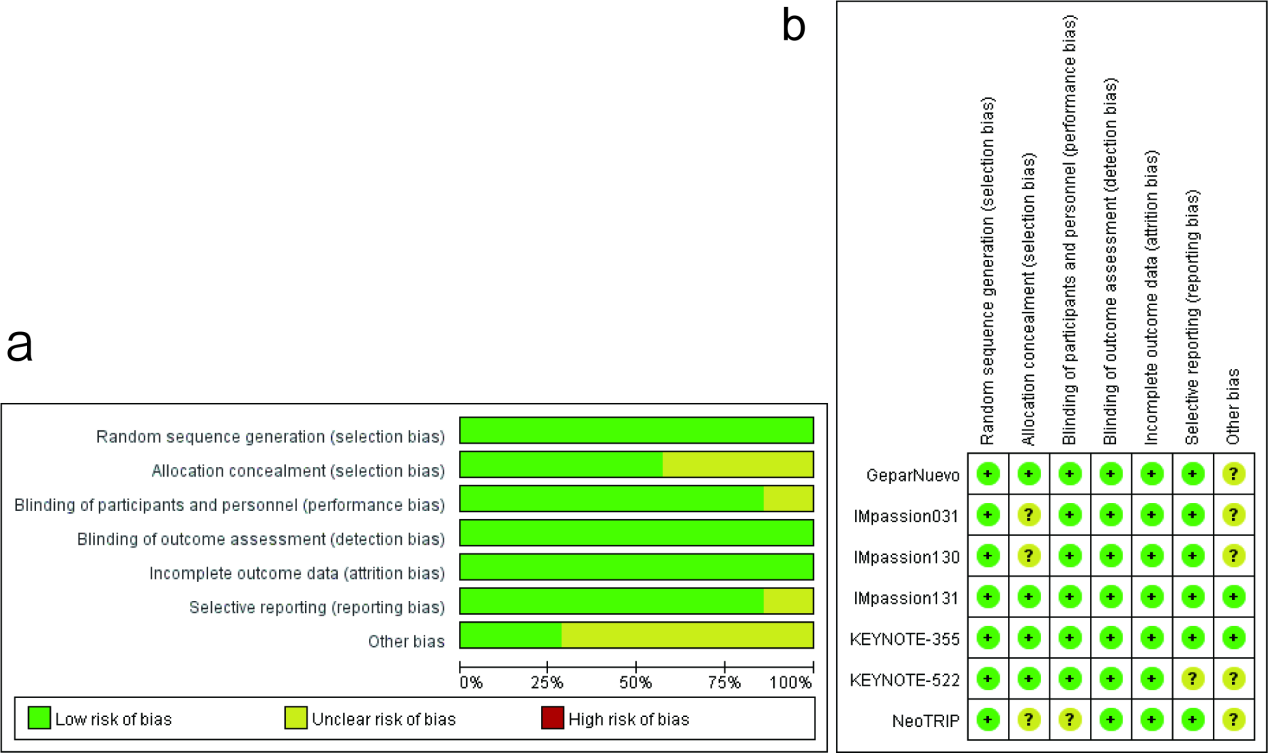


**Supplementary Figure S1. Risk of bias assessment of included studies**

(a): Risk of bias graph. (b): Risk of bias summary: Assessment of the nine included studies for risk of bias items (" + ": low risk of bias; “? ": unclear risk of bias; " − "：high risk of bias")


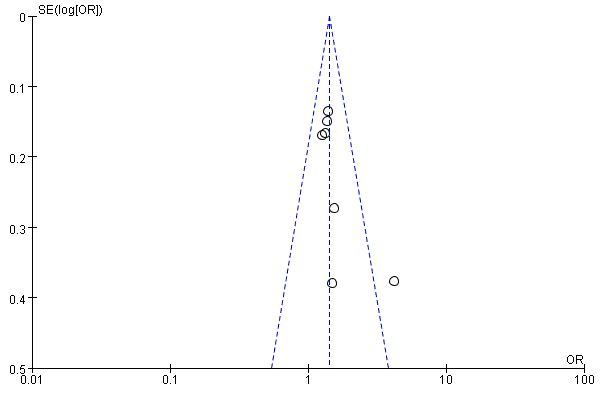


**Supplementary Figure S2. [Funnel plot](javascript:;) of publication bias regarding grades 3-5 AEs results of ICI combined with CT and CT alone**

**Supplementary Table S1. Sensitivity analysis of studies included**

|  | **Fixed effect model** | **Random effect model** |
| --- | --- | --- |
| Total PFS | 0.82 (0.74-0.90) | 0.82 (0.74-0.90) |
| PFS (PD-L1 positive) | 0.68 (0.58-0.79) | 0.67 (0.58-0.79) |
| PFS (PD-L1 negative) | 0.95 (0.82-1.09) | 0.95 (0.82-1.09) |
| Total OS | 0.92 (0.82-1.04) | 0.92 (0.82-1.02) |
| OS (PD-L1 positive) | 0.79 (0.55, 0.97) | 0.81 (0.63-1.04) |
| OS (PD-L1 negative) | 1.01 (0.87-1.17) | 1.01 (0.87-1.17) |
| Total pCR | 1.62 (1.30-2.01) | \| 1.62 (1.30-2.01) \| \| --- \| |
| pCR (PD-L1 positive) | 1.70 (1.30-2.23) | 1.70 (1.30-2.23) |
| pCR (PD-L1 negative) | 1.52 (1.01-2.27) | 1.52 (1.01-2.27) |
| Total ORR | \| 1.35 (1.14- 1.60) \| \| --- \| | 1.35 (1.14- 1.60) |
| ORR (PD-L1 positive) | \| 1.70 (1.24- 2.23) \| \| --- \| | 1.70 (1.24- 2.23) |
| Grades 3-5 AEs | \| \| 1.43 (1.24-1.64) \| \| --- \| \| \| --- \| --- \| | 1.45 (1.21-1.74) |
| Severe AEs | \| 1.53 (1.24-1.87) \| \| --- \| | 1.56 (1.14-2.14) |
| Subgroup analysis total OS | 0.71 (0.53-1.43) | 0.71 (0.53-1.43) |
| Subgroup analysis OS(PD-L1 negative) | 0.84 (0.73-1.96) | 0.81 (0.63-1.03) |

Notes: PFS/OS: HR, 95%CI (Treatment group vs. control group); pCR/ORR: RR, 95%CI (Treatment group vs. control group).

**Supplementary Table S2. Sensitivity analysis regarding grades 3-5 AEs results of ICI combined with CT and CT alone**

| Excluded study | Odds ratio | 95% CI | *I*², χ² *P*-value | *P*-value |
| --- | --- | --- | --- | --- |
| / | 1.45 | 1.24-1.64 | 35%, < 0.00001 | *P* < 0.00001 |
| IMpassion130 | 1.44 | 1.23-1.70 | 46%, < 0.00001 | *P* < 0.00001 |
| KEYNOTE-355 | 1.47 | 1.26-1.71 | 42%, < 0.00001 | *P* < 0.00001 |
| IMpassion131 | 1.45 | 1.25-1.69 | 44%, < 0.00001 | *P* < 0.00001 |
| KEYNOTE-522 | 1.45 | 1.24-1.69 | 45%, < 0.00001 | *P* < 0.00001 |
| IMpassion031 | 1.36 | 1.18-1.57 | 0%, < 0.00001 | *P* < 0.0001 |
| NeoTRIP | 1.43 | 1.24-1.64 | 46%, < 0.00001 | *P* < 0.00001 |
| GeparNuevo | 1.42 | 1.23-1.64 | 45%, < 0.00001 | *P* < 0.00001 |
